# Drug-Resistant Epilepsy: Experience From a Tertiary Care Center in Saudi Arabia

**DOI:** 10.7759/cureus.61913

**Published:** 2024-06-07

**Authors:** Adilah Alturaifi, Hatoon Alshaikh, Osama Khojah, Abdulaziz Alqarni, Tarfah Albedaiwi, Amira Albluwi, Elaf Alqurashi, Husun Kecheck, Halah Fallatah, Reuof Almakati, Raghad Gahtani, Rahaf Aljohani, Madihah Alhubayshi, Seraj Makkawi

**Affiliations:** 1 Department of Neuroscience, Ministry of National Guard Health Affairs, Jeddah, SAU; 2 Department of Research and Development, King Abdullah International Medical Research Center, Jeddah, SAU; 3 College of Medicine, King Saud Bin Abdulaziz University for Health Sciences, Jeddah, SAU

**Keywords:** antiseizure medication, intractable epilepsy, refractory epilepsy, drug-resistant epilepsy, epilepsy

## Abstract

Objectives

This study aimed to describe the clinical characteristics, investigational results, and management strategies in patients with drug-resistant epilepsy (DRE).

Methods

This retrospective cohort study included all adult and adolescent patients (aged 14 years or older) diagnosed with DRE who visited the adult neurology clinic at King Abdulaziz Medical City, Jeddah, Saudi Arabia from January 2019 to December 2021. DRE was defined as failure to achieve seizure freedom despite undergoing adequate trials of two well-tolerated and appropriately selected antiseizure medications.

Results

This study included 299 patients with DRE. Most patients were in their second to fourth decade, with a mean age of 37 ± 17 years. Focal onset epilepsy was diagnosed in 52.5% of the patients, and an etiology for epilepsy was determined in 44.1% of the patients. Findings in brain magnetic resonance imaging were abnormal in 49% of the patients, whereas abnormal findings in electroencephalograms were found in 27.5%. The most common antiseizure medication was levetiracetam (67.6% of cases).

Conclusion

The findings of this study confirm the challenges in diagnosing and managing patients with DRE and emphasize the necessity for careful and comprehensive patient evaluation. Further research is needed to investigate the effectiveness, safety, and accessibility of diagnostic and therapeutic resources for patients with DRE.

## Introduction

Epilepsy is one of the most common neurological disorders worldwide, with an estimated lifetime prevalence of 15.4 per 1000 individuals [1]. It also accounts for the highest disability-adjusted life year rates among neurological diseases in both men and women [2]. In Saudi Arabia, the estimated prevalence of epilepsy is 6.54 per 1000 individuals [3]. People with epilepsy (PwE) are at high risk of developing drug-resistant epilepsy (DRE), with newly diagnosed patients having a 20-40% chance of remaining refractory to antiseizure medication [4]. Drug resistance has a strong impact on the quality of life (QoL) of PwE. In general, major depressive disorders and other mood and anxiety disorders are highly prevalent in PwE, which directly affects QoL compared to that of the general population [5,6]. Patients with DRE have lower QoL scores and greater social difficulties and psychiatric comorbidities than other PwE [7-9]. Additionally, people with DRE experience longer episodes of seizures and more frequent episodes of status epilepticus, cognitive decline, worsening co-morbidities, and an increased risk of sudden unexpected death [10]. Evidence indicates that surgical treatment for DRE markedly improves patient outcomes [11]. However, less than 1% of the patients with DRE are referred for surgery, with a referral delay for candidate patients being, on average, over 20 years [7]. DRE presents an ongoing challenge for clinicians, researchers, and patients alike, compounded by a diverse and heterogeneous patient population and numerous proposed hypotheses regarding its underlying mechanisms [12]. The aim of this study was to describe the local experience with patients with DRE, including possible etiologies, clinical characteristics, limitations of surgical referral, and the burden of frequent hospitalization in our centers.

## Materials and methods

This retrospective cohort study used a consecutive sampling technique to include all adult and adolescent patients (≥14 years) diagnosed with DRE who visited the neurology clinic from January 2019 to December 2021. The study was conducted at King Abdulaziz Medical City in Jeddah, Saudi Arabia, which is a tertiary hospital with a specialized epilepsy clinic and served nearly 2000 patients with epilepsy over a three-year period. The adult neurology clinic at King Abdulaziz Medical City, similar to the practices at other hospitals in Saudi Arabia, follows patients from the age of 14 years. In our cohort, only 5% (n = 15) of the patients were younger than 18 years.

DRE was defined as “failure of adequate trials of two tolerated, appropriately chosen and used antiepileptic drug schedules (whether as monotherapies or in combination) to achieve sustained seizure freedom” [13]. During the diagnostic phase, the assessment for DRE included but was not limited to detailed clinical history, seizure semiology, routine electroencephalography (EEG) monitoring, and neuroimaging. However, it lacked long-term video EEG monitoring. Additional investigations were performed on a case-by-case basis to ascertain the etiology. The etiology was determined to be secondary to CNS infection only after the patients were evaluated in the neurology clinic and not during the acute phase of the infection.

The surgical referral process at our center is generally structured as follows: after evaluating the patients for surgical referral, a meeting is held between an epileptologist and a neurosurgeon to assess the benefits of surgical intervention. A decision is then made to either continue follow-up at our institution's neurosurgery clinic, which provides vagal nerve stimulation, or to refer the patient to other tertiary care centers that offer more advanced surgical options.

The electronic medical records of all patients diagnosed with epilepsy were reviewed to include those with DRE. The collected data included patient demographics, comorbidities, disease characteristics, neurological examination findings, results from brain magnetic resonance imaging (MRI) and EEG, antiseizure medication (ASM) used, ASM side effects, and surgical referrals. The data were entered into SPSS version 20 (IBM Corp., Armonk, NY) for statistical analysis. The quantitative variables are presented as mean ± standard deviation and the qualitative variables as frequency and percentages.

## Results

The study included a total of 299 patients with DRE who visited our neurology clinic from 2019 to 2021. The mean age was 37 ± 17 years (ranging from 15 to 102 years) and the sex distribution was equal. Over the three-year period, the median number (range) of clinical and emergency room (ER) visits and admissions to the intensive care unit (ICU) were four (0-22), 0 (0-21), and 0 (0-4), respectively. The neurological examination findings were unremarkable in more than half of the participants (57.9%), and focal onset epilepsy was the most common type (52.5%). Among the patients, 130 (43.5%) presented with one or more associated comorbidities, with intellectual disability, hypertension, and diabetes mellitus being the most frequent. Etiologies for epilepsy, such as developmental disorders (e.g., congenital brain malformation), brain tumors, and trauma were identified in 132 (44.1%) patients. The participants’ demographics and clinical characteristics are presented in Table 1.

**Table 1:** Baseline demographics and clinical characteristics of the patients (n = 299). SD: standard deviation; MDD: major depressive disorder; CKD: chronic kidney disease; CAD: coronary artery disease; COPD: chronic obstructive pulmonary disease; CNS: central nervous system.

Variable	Frequency (%) or mean (± SD)
Age: mean ± SD	37 ± 17
Sex: female	151 (50.5%)
Family history of epilepsy	12 (4%)
History of febrile convulsions	6 (2%)
Epilepsy type	
Generalized	86 (28.8%)
Focal	157 (52.5%)
Unknown	56 (18.9%)
Comorbidities	
Intellectual disability	41 (13.7%)
Hypertension	36 (12%)
Diabetes mellitus	32 (10.7%)
Stroke	22 (7.4%)
Hypothyroidism	19 (6.4%)
MDD	19 (6.4%)
Anxiety	16 (5.4%)
Dyslipidemia	11 (3.7%)
Cerebral palsy	8 (2.7%)
Migraine	8 (2.7%)
Asthma	7 (2.3%)
CKD	6 (2%)
Liver disease	4 (1.3%)
CAD	3 (1%)
COPD	1 (0.3%)
Dementia	1 (0.3%)
Bipolar disorder	1 (0.3%)
Epilepsy etiologies	
Unknown	167 (55.9%)
Developmental disorders	30 (10%)
Vascular	21 (7%)
Mesial temporal sclerosis	16 (5.4%)
Trauma	15 (5%)
Brain tumor	14 (4.7%)
CNS infection	11 (3.7%)
Immune-mediated	5 (1.7%)
Other	20 (6.7%)

**Figure 1:**
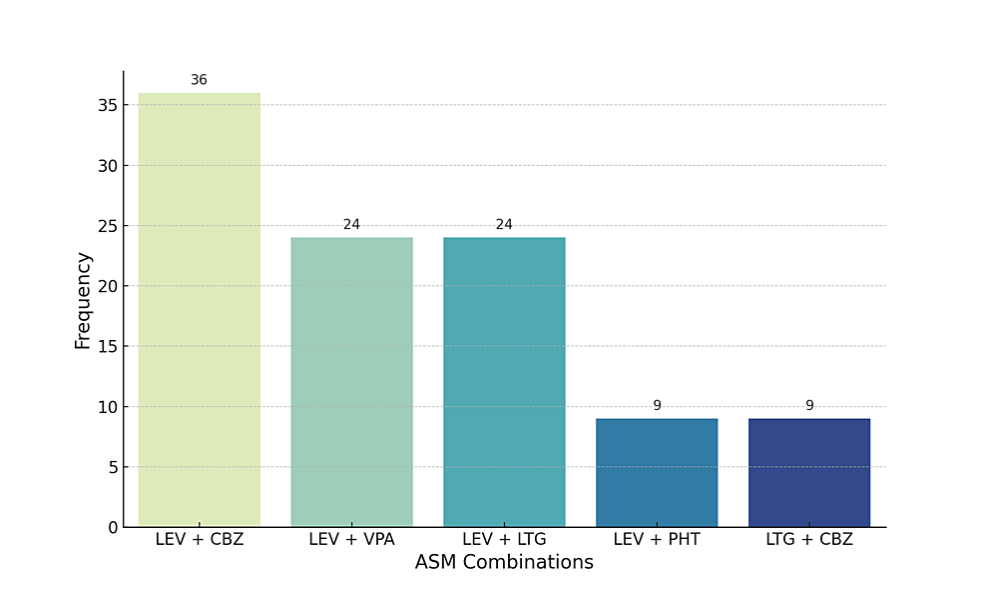
Common antiseizure medication combinations utilized in patients with drug-resistant epilepsy. ASM: antiseizure medication; LEV: levetiracetam; CBZ: carbamazepine; VPA: valproic acid; LTG: lamotrigine; PHT: phenytoin.

The median number of ASMs used per patient was two (IQR = 1). As can be seen in Table 2, the included patients were prescribed a wide array of ASMs, of which levetiracetam (LEV) was the most commonly used (67.6% of cases). A total of 127 patients (42.5%) discontinued or switched ASMs; of those patients, 94.5% experienced uncontrolled seizures, prompting the switch in ASM. Undesirable side effects were the reason for discontinuing or switching ASMs in 26.8% of patients, while family planning was the reason for 0.8% of patients. Side effects that led to discontinuation or switching included mood disorders (29.4%), skin rashes (14.7%), drowsiness (11.8%), sleepiness (11.8%), abnormal levels of liver enzymes (8.8%), complete blood count abnormalities (5.9%), electrolyte imbalance (5.9%), weight gain (5.9%), and gastrointestinal symptoms (5.9%). Of the numerous combinations of ASMs, the five most common, which were administered to 34% of the patients, are depicted in the graph in Figure 1.

**Table 2:** Antiseizure medications (ASMs) used by patients with drug-resistant epilepsy (n = 299).

ASMs	Frequency (%) of active ASMs	Frequency (%) of discontinued ASMs
Levetiracetam	202 (67.6%)	26 (11.4%)
Carbamazepine	109 (36.5%)	13 (10.7%)
Lamotrigine	103 (34.4%)	18 (14.9%)
Valproic acid	87 (29.1%)	17 (16.3%)
Lacosamide	73 (24.4%)	14 (16%)
Topiramate	41 (13.7%)	10 (19.6%)
Phenytoin	35 (11.7%)	10 (22.2%)
Clonazepam	28 (9.4%)	6 (17.6%)
Oxcarbazepine	22 (7.4%)	3 (12%)
Clobazam	19 (6.4%)	5 (20.8%)
Phenobarbital	9 (3%)	2 (18.2%)
Gabapentin	5 (1.7%)	0 (0%)
Vigabatrin	3 (1%)	1 (25%)
Perampanel	2 (0.7%)	0 (0%)
Lorazepam	1 (0.3%)	1 (50%)
Pregabalin	1 (0.3%)	1 (50%)

Brain MRI was performed in 70.2% of the patients, revealing abnormalities in 49%. Abnormalities included encephalomalacia (20.4%), mesial temporal sclerosis (16.5%), brain infarction (12.6%), brain atrophy (11.7%), brain tumor (11.7%), hydrocephalus (5.8%), vascular malformation (4.9%), brain infection (e.g., brain abscess or sequelae of bacterial meningitis or viral encephalitis) (4.9%), brain hemorrhage (2.9%), and brain dysplasia (2.9%). Mesial temporal sclerosis was seen equally in the left or right lobe in 41.2% of patients, while it was detected bilaterally in 11.7% of patients. Brain tumors commonly affected the frontal lobe (25%), parietal lobe (16.7%), or temporal lobe (16.7%), with involvement of more than one lobe in 41.7% of the patients.

EEG was available for review in 78.9% of the patients, and abnormalities appeared in 27.5%. Among those with abnormalities, 35.4% of the patients exhibited focal slowing and 24.6% exhibited generalized slowing. Focal and generalized epileptiform discharges were present in 55.4% and 29.2% of the patients, respectively. Forty-three patients (14.4%) were referred for epilepsy surgery.

## Discussion

Despite considerable advances in epilepsy management, the disorder continues to impose a substantial burden in terms of morbidity and mortality [8]. This retrospective study examined a cohort of 299 patients with DRE and outlined their clinical profiles, ASM use, EEG and MRI findings, and surgical referrals. Two-thirds of the patients received a combination of LEV with one or more ASMs, with LEV and carbamazepine (CBZ) being the most commonly used regimen. Approximately less than half of the patients discontinued or switched ASM due to inadequate control or side effects. Etiologies for epilepsy were identified in nearly half of the participants; most had comorbidities, and a small subset was referred for surgery. These findings underscore the complex clinical profiles and management challenges in patients with DRE.

Common DRE etiologies include cortical dysplasia, distal brain injury, and tuberous sclerosis, which affect patients at a young age. Consequently, the mean age of patients with DRE is shifted to between the second and fourth decades of life, resulting in a higher prevalence in younger populations [14-17]. Regarding the types of epilepsy, focal epilepsy was predominant, which is in line with the results from earlier studies [18-20]. Additionally, almost half of the patients in our sample had one or more associated comorbidities (43.5%); this finding is consistent with the results of other studies [21,22] and is particularly relevant as the presence of a comorbid disease can affect epilepsy management and vice versa. Systemic comorbidities and their treatment may lower the seizure threshold and change the metabolism and excretion of ASMs, as seen, for instance, in cases of renal or hepatic failure [22]. It is important to emphasize that these concurrent health conditions may adversely affect overall QoL.

Several studies have indicated that symptomatic etiologies strongly predict drug resistance in patients with epilepsy [23,24], with structural etiologies being the most frequently reported in patients with DRE [23,25]. Similarly, in our patients with a known etiology of epilepsy, structural etiologies were the most common. A 2019 meta-analysis of cohort studies [26], aimed at identifying risk variables for DRE, found that abnormal EEG, symptomatic etiology, febrile seizures, and multiple seizure types constitute the main related risk factors. These risk factors were similarly present in our sample, with developmental disorders being the most frequent. The cause of epilepsy was unidentified in more than half of our cohort highlighting a substantial gap in the thorough assessment of etiologies. A thorough etiological evaluation may include but is not limited to genetic, metabolic, and autoimmune testing and advanced neuroimaging techniques. Although these tests were available and performed in some patients, assessing for structural causes through neuroimaging was done more frequently.

According to the recommendations of the International League Against Epilepsy (ILAE), all patients with epilepsy should undergo EEG and brain MRI unless contraindicated [27,28]. EEG was available for review in approximately 80% of our patients; similar rates have been reported in the literature [20]. A study investigating electrophysiological predictors for DRE revealed that abnormal EEG findings were significantly more common in patients with DRE (69.2%) than in the well-controlled group (44.7%) [23]. In contrast, in our cohort, abnormal EEG findings were reported in only 27.5% of those who underwent EEG. This discrepancy may stem from variations in the EEG recording timing, treatment responses, heterogeneity of underlying epilepsy etiologies, as well as differences in study populations and methodologies. The low rate of brain MRI utilization among our patients (approximately two-thirds of the patients) compared to the rates reported in recent studies (83-93% for participants with DRE) raises important considerations [29,30]. Possible contributing factors include issues related to patient awareness, compliance, and resource constraints. At our institution, where services are provided free of charge, financial barriers such as cost and accessibility are unlikely to be the primary cause. Among those who underwent brain MRI, the most common abnormalities were encephalomalacia (20.4%), followed by mesial temporal sclerosis (16.5%). Similar results were obtained in a previous cohort study, although mesial temporal sclerosis and encephalomalacia were the most common (26.9%) and second most common (10.7%) MRI findings, respectively [31]. To accurately determine seizure origin, it is imperative to supplement MRI findings with other clinical findings and tests, such as routine EEG or long-term video EEG monitoring [31]. Evidently, comprehensive imaging evaluation should be considered in patients with DRE, as targeted interventions, such as surgery and deep-brain stimulation, which can reduce seizure frequency or confer seizure freedom, are critically reliant on the identification of epileptogenic zones [32]. It is of utmost importance for all patients with epilepsy, particularly those with DRE, to undergo EEGs and brain MRIs as recommended by ILAE. The underutilization of these diagnostic tests in our cohort reflects a gap in adhering to established guidelines. Performing advanced investigations, utilizing treatment options, and surgical referrals could be influenced or guided by the results of these tests. These findings should prompt further research into identifying barriers to the optimal use of diagnostic tools in epilepsy care, such as logistical, financial, or due to a lack of awareness among healthcare providers and/or patients.

In terms of ASM use, most of our patients were on LEV at the time of the study. Notably, the selection of ASMs depends on various factors, including patient age, epilepsy type, epilepsy syndrome, concomitant medications, and safety, tolerability, and efficacy of the ASMs. For DRE, experts recommend rational polytherapy, which seeks ASM combinations that work synergistically to improve efficacy without increasing adverse effects [33]. The most common ASM combinations used in our cohort were LEV and CBZ, LEV and valproic acid (VPA), and LEV and lamotrigine (LTG). According to a 2023 scoping review of rational polytherapy in patients with DRE [34], the combination of VPA and LTG is promising, despite the known drug interaction that requires slow titration of the latter. However, the optimal combinations of ASMs for DRE remain unclear and are largely determined on a case-by-case basis. Certain ASM combinations can increase the risk of undesirable effects; for example, the likelihood of developing tremors may be increased with a combination of VPA and LTG [35]. Both drugs, independently or in combination, can lead to postural and action tremors; therefore, the decision to reduce the dose of a specific drug depends on the individual therapeutic effects [35]. Among second-generation ASMs, LEV could be a crucial component in ASM polytherapy due to its minimal pharmacological interactions [36]. A comprehensive discussion about potential ASM options and their side effects should be conducted with patients, who should then be closely followed and monitored for side effects.

In this study, we also assessed patients with DRE who were referred for surgery and noted that only 14.4% of the patients were referred for surgical evaluation. The reasons for not undergoing a surgical evaluation generally vary and can be related to the physician or the patient, such as experience of infrequent or mild seizures, generalized epilepsy syndromes, or the presence of multifocal lesions on MRI [37]. It is important to note that during the duration of the study, our center did not offer epilepsy surgery but did refer patients to other capable centers. Performing this study in a center that offers epilepsy surgery might yield different results. Previous studies have shown a 20-year delay for the average patient with DRE to be referred to an epilepsy surgery center [38,39], although delaying surgery may reduce the likelihood of postoperative seizure freedom [40].

Several limitations of this study warrant acknowledgment. First, the study was conducted in a single center, which may constrain the generalizability of the findings to other settings. Second, reliance on retrospective data can introduce documentation errors or recall bias. Third, this study was conducted in a center that did not provide comprehensive epilepsy surgery services during most of the duration of the study. Fourth, we were not able to independently assess and exclude psychogenic non-epileptic seizures (PNES). However, cases suspected to have or diagnosed with PNES were excluded. Lastly, the study did not assess the impact of various treatments on patient outcomes, which could have yielded valuable insights regarding the effectiveness of different therapeutic approaches.

## Conclusions

This study highlights the clinical and diagnostic characteristics of patients with DRE at a single center. We found that a considerable proportion of patients with DRE had comorbidities and abnormal findings on MRI and EEG, underscoring the necessity for comprehensive evaluations and individualized treatment plans. Based on these findings, we propose several recommendations. First, healthcare providers should perform exhaustive assessments of patients with DRE to identify underlying etiologies, comorbidities, and abnormal diagnostic findings that can affect patient outcomes. Second, physicians should consider surgical referrals for eligible patients to improve seizure control and QoL. Third, additional research is required to investigate the effectiveness and accessibility of different diagnostic and treatment modalities, including surgery, pharmacotherapy, and non-pharmacological interventions, in patients with DRE. Lastly, efforts should be made to improve patient education and compliance to enhance treatment adherence and minimize the risk of adverse outcomes.
